# Melanoma Classification Using a Novel Deep Convolutional Neural Network with Dermoscopic Images

**DOI:** 10.3390/s22031134

**Published:** 2022-02-02

**Authors:** Ranpreet Kaur, Hamid GholamHosseini, Roopak Sinha, Maria Lindén

**Affiliations:** 1 School of Engineering, Computer and Mathematical Sciences, Auckland University of Technology, Auckland 1010, New Zealand; hamid.gholamhosseini@aut.ac.nz (H.G.); roopak.sinha@aut.ac.nz (R.S.); 2 School of Innovation, Design and Engineering, Mälardalen University, 722 20 Västerås, Sweden; maria.linden@mdh.se

**Keywords:** skin cancer, melanoma, classification, deep convolutional neural networks

## Abstract

Automatic melanoma detection from dermoscopic skin samples is a very challenging task. However, using a deep learning approach as a machine vision tool can overcome some challenges. This research proposes an automated melanoma classifier based on a deep convolutional neural network (DCNN) to accurately classify malignant vs. benign melanoma. The structure of the DCNN is carefully designed by organizing many layers that are responsible for extracting low to high-level features of the skin images in a unique fashion. Other vital criteria in the design of DCNN are the selection of multiple filters and their sizes, employing proper deep learning layers, choosing the depth of the network, and optimizing hyperparameters. The primary objective is to propose a lightweight and less complex DCNN than other state-of-the-art methods to classify melanoma skin cancer with high efficiency. For this study, dermoscopic images containing different cancer samples were obtained from the International Skin Imaging Collaboration datastores (ISIC 2016, ISIC2017, and ISIC 2020). We evaluated the model based on accuracy, precision, recall, specificity, and F1-score. The proposed DCNN classifier achieved accuracies of 81.41%, 88.23%, and 90.42% on the ISIC 2016, 2017, and 2020 datasets, respectively, demonstrating high performance compared with the other state-of-the-art networks. Therefore, this proposed approach could provide a less complex and advanced framework for automating the melanoma diagnostic process and expediting the identification process to save a life.

## 1. Introduction

Skin cancer is an invasive disease caused by the abnormal growth of melanocyte cells in the body, which tend to replicate and spread through lymph nodes to destroy surrounding tissues [1]. The damaged cells develop a mole on the external skin layer, categorized as malignant or benign, whereas melanoma is considered cancer because it is more dangerous and life-threatening. Skin cancer is a widespread and dangerous disease globally, with 300,000 newly diagnosed cases and over 1 million deaths each month worldwide in 2018 [2]. Melanoma is more prevalent globally, becoming the 19th most common disease with the highest mortality rate [2]. As per the statistics of the International Agency for Research for Cancer (IARC) [3], 19.3 million new cases were diagnosed with cancer, with a mortality rate of about 10 million people in 2020. Moreover, the number of new cases found in the United States were 100,350, and the number of people who died in 2020 were approximately 6850. According to the American Cancer Society [4], 106,110 new melanoma cases were predicted to be diagnosed (nearly 62,260 in men and 43,850 in women) and about 7180 melanoma patients were estimated to die in 2021. Some environmental and genetic factors such as fair complexion, pollution, family history, and sunburn may lead to the formation of skin cancer. The control over mortality rate due to cancer is challenging; however, the latest development in image processing and artificial intelligence approaches may help diagnose melanoma early as early detection and prognosis can increase the survival rate. Moreover, computer-aided diagnostic (CAD) tools save time and effort compared with existing clinical approaches.

During diagnosis, an expert dermatologist performs a series of steps, starting with a visual inspection of a skin lesion by the naked eye; then dermoscopy, which is a magnifying lens to view lesion patterns in detail; and finally, a biopsy [5]. These conventional methods are time-consuming, expensive, and laborious. Achieving an accurate diagnosis is entirely subjective depending upon the expert’s skillset, resulting in variations in their predictions. Many experts analyze lesions based on the ABCDE [6] metrics, which define the asymmetry, border, color, diameter above 6 mm, and evolution over time. However, it requires intensive knowledge and proficiency that might not be available in clinical settings. It is found that the accuracy of correctly identifying skin lesions by a dermatologist is less than 80% [7]. Additionally, there is a limited number of expert dermatologists available globally in the health sector.

To diagnose a skin lesion at the earliest stage and to solve the complexities mentioned above, comprehensive research solutions have been proposed in the literature using computer vision algorithms [8]. The classification methods vary, including decision trees (DT) [9], support vector machines (SVM) [10], and artificial neural networks (ANN) [11]. A detailed review of these methods is explained in the paper in Reference [12]. Many machine learning methods have constraints in processing data, such as requiring high contrast, noise-free, and cleaned images that do not apply in the case of skin cancer data. Moreover, skin classification depends on features such as color, texture, and structural features. The classification may lead to erroneous results with poor feature sets as skin lesions consist of a high degree of inter-class homogeneity and intra-class heterogeneity [13]. The traditional approaches are parametric and require training data to be normally distributed, whereas skin cancer data is uncontrolled. Each lesion consists of a different pattern; thus, these methods are inadequate. For these reasons, deep learning techniques in skin classification are very effective in assisting dermatologists in diagnosing lesions with high accuracy. Several detailed surveys elaborate on the application of deep learning in medical applications [14].

**Figure 1:**
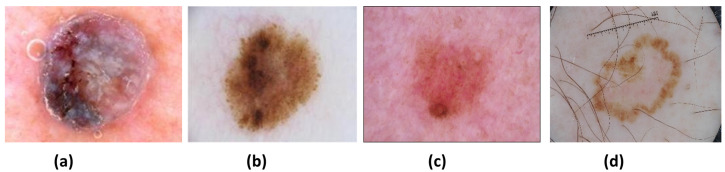
Different types of skin lesions: (**a**) MEL, (**b**) BEN, (**c**) NV, and (**d**) SK.

There are mainly three types of skin cancer: basal, squamous, and melanocyte [15]. The most commonly occurring type of cancer, basal cell carcinoma, grows very slowly and does not spread to other parts of the body. It tends to recur, so eradicating it from the body is important. Squamous cell carcinoma is another type of skin cancer that is more likely to spread to other body parts than basal cell carcinoma and penetrates deeper into the skin. Melanocytes, the cells involved in the last type, produce melanin when exposed to sunlight, giving the skin its brown or tan color. The melanin in these cells protects the skin from sunlight, but if it accumulates in the body, it forms cancerous moles, also known as melanoma cancer. Based on their tendency to cause minimal damage to surrounding tissues, basal and squamous cancers are considered benign, whereas melanocyte-based cancers are considered malignant and can be life-threatening. The most popular datasets employed in this work is from the International Skin Imaging Collaboration (ISIC) [16], which contains different skin lesions. There are mainly four types of lesions (see Figure 1) in the ISIC 2016, 2017, and 2020 data: (a) Nevus (NV), (b) Seborrheic keratosis (SK), (c) Benign (BEN) (d) Melanoma (MEL). NV cancer has distinct edges that primarily appear on the arms, legs, and trunk in pink, brown, and tan colors. Next is the SK, of which its non-cancerous appearance is waxy brown, black, or tan colors. Another non-cancerous lesion type is BEN, which does not invade surrounding tissues or spread into the body. Both NV and SK lesion types are considered BEN. Lastly, MEL is a large brown mole with dark speckles; it sometimes bleeds or changes color over time. It is a dangerous type of cancer that quickly spreads to other organs of the body. MEL is further divided into many types: acral, nodular, superficial, and lentigo. This research aims to identify and distinguish between MEL and BEN cancers.

Although deep learning approaches are highly effective in processing complex data, skin classification is still a challenging task due to a few reasons:

(a) Skin lesion classes in given datasets are highly imbalanced. For example, NV contains more samples than SK and MEL in the ISIC 2017 set, and BEN samples are more common than MEL in the ISIC 2020 set.
(b) Lesions contain noisy artefacts such as hairlines, gel bubbles, ruler marks, and poor contrast.
(c) Lesion types are difficult to distinguish due to high intra-class differences and inter-class similarities.

Moreover, there have been a few challenges during the design of classification approaches, such as (a) achieving a high prediction rate despite the class imbalance problem, (b) less complex and lightweight network architectures, and (c) low inference time. Popular deep learning pre-trained networks cannot be applied to skin cancer problems in general, as those networks are trained on different datasets such as ImageNet. Hence, the proposed research aims to develop, implement, and evaluate a deep learning-based, highly efficient network for melanoma vs. benign classification. The contributions of the proposed work are as follows:

- A new design of the DCNN model for classifying skin lesions as malignant or benign on dermoscopic images is proposed by building multiple connected blocks to allow for large feature information to flow directly through the network.
- The depth of the network is optimized by conducting several experimental trials on the validation set by repeating sub-blocks with some specific ratio to form a deep neural network.
- Each block of the network uses different parameters such as the number of kernels, filter size, and stride to extract low- and high-level feature information from lesions.
- The proposed model achieves higher performance than other state-of-the-art methods on the adopted ISIC datasets, with fewer filters and learnable parameters. Thus, it is a lightweight network for classifying a large skin cancer dataset.

## 2. Related Work

Skin cancer is prevalent around the world, becoming the cause of a large number of deaths each year [17]. It is an aggressive disease; thus, it is vital to perform early detection to save lives. Clinical experts visually observe lesions based on the ABCDE [6] criteria followed by some histopathological tests. For automation of the classification process, several artificial intelligence-based algorithms have been proposed that comprise the standard phases such as preprocessing, feature extraction, segmentation, and classification. Many classification approaches [18,19] were highly dependent upon handcrafted feature sets, which have low generalization capability for dermoscopic skin images due to a deep understanding of biological patterns. Lesions have a substantial visual resemblance and are highly correlated because of their similarity in colors, shape, and size leading to poor feature information [20]. Thus, handcrafted feature-based approaches are not suitable for skin classification problems. The advantage of deep learning techniques is that they can be directly applied to classification without any preprocessing phase. Deep networks are efficient at calculating detailed features to perform accurate lesion classification compared with shallow networks. The first breakthrough of applying DCNN on skin cancer came from Esteva et al. [5] used a pre-trained Inceptionv3 model on 129,450 clinical images to perform classification in 2032 different diseases. Their network was compared against 21 board-certified medical experts to perform binary classification between the two deadliest skin cancers: malignant and nevus. Experts testified that the proposed network could identify skin cancer with high performance. Another work by Y. Li et al. [21] proposed a lesion index calculation unit (LICU) that computes heat maps to filter coarse classification outcomes from the FCRN model. This unit measures the contribution of each pixel from the segmented map towards classification. The framework was evaluated on the ISIC 2017 dataset. J. Zhang et al. [22] proposed a CNN implementing an attention residual learning (ARL) for skin classification consisting of multiple ARL blocks followed by global average pooling and classification layers.

The network explored the intrinsic self-attention ability of a deep convolutional neural network (DCNN). Each ARL block uses a residual learning mechanism and generates attention maps at lower layers to improve classification performance. Iqbal et al. [23] designed a DCNN model for multi-class classification of a skin lesion on the ISIC 2017-19 datasets. Their model consists of multiple blocks connected to pass feature information from top to bottom of the network utilizing 68 convolutional layers. Similarly, Jinnai et al. [24] employed faster region-based CNN (FRCNN) to classify melanoma from 5846 clinical images rather than dermoscopy. They manually created bounding boxes for lesion regions to prepare the training dataset. The FRCNN outperformed ten board-certified dermatologists and ten dermatology trainees, providing higher accuracy.

An investigation on increasing the performance of the model in terms of the area under the curve (AUC), accuracy, and other metrics by creating ensemble CNN models was proposed by Barata et al. [18]. The output from the classification layers of four different networks, such as GoogleNet, AlexNet, VGG, and ResNet, was fused to form an ensemble model for three class classifications. Jordan Yap et al. [25] proposed a method that considers several image modalities, including patient’s metadata, to improve the classification results. The ResNet50 network was differently applied over dermoscopic and macroscopic images, and their features were fused to perform the final classification. Their multimodel classifier outperformed the basic model using only macroscopy with an AUC of 0.866. Similarly, Gessert et al. [26] presented an ensemble model designed from EfficientNets, SENet, and ResNeXt WSL to perform a multi-class classification task on the ISIC 2019 dataset. They applied a cropping strategy on images to deal with multimodel input resolutions. Moreover, a loss balancing approach was implemented to tackle imbalanced datasets. Srinivasu et al. [27] presented a DCNN based on MobileNetV2 and Long Short-Term Memory (LSTM) for lesion classification on the HAM10000 dataset. Compared with other CNN models, MobileNetV2 offered advantages in terms of a low computational cost, a reduced network size, and compatibility with mobile devices. The LSTM network retained timestamp information about the features calculated by MobileNetV2. The use of LSTM with MobileNetV2 enhanced the system accuracy to 85.34%.

Another method was a Self-supervised Topology Clustering Network (STCN) given by Wang. et al. [28] to classify unlabelled data without requiring any prior class information. The clustering algorithm was used to organize anonymous data into clusters by maximizing modularity. Features learned at different levels of variations such as illumination, point of view, and background were considered by the STCN model. Some studies [29,30] utilized pre-trained networks such as Xception, AlexNet, VGGNet, and ResNet using transfer learning and compared their performance. The fully connected layers were changed to use existing networks for skin lesion classification, and hyperparameters are required to fine-tune to achieve the best performance. The systematic review articles in [14,31] can be referred for detailed insights of deep learning approaches used for skin cancer classification. The detailed survey article in [32] explained the possible solution to automatic skin cancer detection system, considered various challenges of skin cancer problems, and provided research directions to be considered for this problem.

## 3. Materials and Methods

### 3.1. Datasets and Splitting

The dermoscopic datasets were obtained from the ISIC 2016 [33], ISIC 2017 [34], and 2020 [35] challenges. The original ISIC 2016 and 2017 datasets contained fewer samples. For example, the ISIC 2016 contained 900 samples with 727 BEN and 173 MEL samples, and thte ISIC contained a total of 2000 samples with 374 MEL, 254 BKL, and 1372 NV samples. The classes in these datasets were highly unbalanced, which can degrade the model’s performance. Therefore, additional samples in each class were added from the ISIC archive [16]. In the new distribution of samples among datasets, there are 1719 samples with two lesion types, BEN and MEL, in the ISIC 2016 dataset. In the ISIC 2017 dataset, there are 4172 lesion samples with different lesions types, such as SK, MEL, and NV. The SK and NV lesions are benign cancer growths; thus, these two types are considered under the BEN lesion type. Furthermore, the total number of images taken in the ISIC 2020 set was 10,070, with two different lesion labels: MEL and BEN. The three datasets ISIC 2016, ISIC 2017, and ISIC 2020 were divided into three subsets: training, validation, and test sets (see Table 1, Table 2 and Table 3). For all of the datasets, 70% of total samples were taken in the training set, 10% was taken in the validation set, and the remaining 20% was provided for the test set. The proportion of samples in the training set was kept higher to provide enough training to the network. The network’s performance was monitored based on validation data for hyper-tuning the parameters. Lastly, the test data were used to evaluate the network’s performance. An additional dataset named $PH^{2}$ [36] from the Dermatology Service of Hospital Pedro Hispano, Matosinhos, Portugal, is employed. This set contained a total of 200 samples, with BEN and MEL lesion types.

### 3.2. Data Normalization

For the ISIC 2016 and 2017 datasets, additional lesion samples were added to balance the distribution. Some skin samples had similar lesion structures but different names, making them difficult to distinguish. Thus, data normalization was applied to eliminate data redundancy and operations such as updation and deletion of anomalies. To remove data duplicacy, first, the image was converted to grayscale as I(x,y) to $I^{\prime }\left(x,y\right)$, and then, its histogram was calculated from the images:

(1) $$h1=histogram\left(I\left(x,y\right)\right),\;h2=histogram\left(I^{\prime }\left(x,y\right)\right)$$

Afterwards, the mean of each image was determined using the average function:

(2) h1=mean(h1),h2=mean(h2)

The correlation index between images was calculated using following equation and was compared. If the correlation between two images was greater than 0.99, the images were considered identical and one copy was discarded. Table 1 and Table 2 show the final distributions of samples used for the experiments after elimination of redundant data.

(3) $$Correlation=\frac{\sum_{x}\sum_{y}\left(I\left(x,y\right)-h1\right)\left(I^{\prime }\left(x,y\right)-h2\right)}{\sqrt{\left(\sum_{x}\sum_{y}{\left(I\left(x,y\right)-h1\right)}^{2}\right)\left(\sum_{x}\sum_{y}{\left(I^{\prime }\left(x,y\right)-h2\right)}^{2}\right)}}$$

### 3.3. Preprocessing Operations

Standard operations were applied to make image samples suitable for processing in preprocessing. First, images were cropped to transform them into square images by locating the lesions in the centre of the image. Each category consists of a varying dimension of image resolution ranging from 576×768 to 1024×1024, with three color channels RGB. Thus, each image was rescaled to 128×128 dimensions using the bilinear interpolation method while preserving their aspect ratio and minimizing the computational cost. There was no need to apply any noise removal method for eliminating hairlines, gel bubbles, and ruler and ink marks because the proposed DCNN model efficiently processes raw images contaminated with artefacts.

### 3.4. Data Augmentation

**Figure 2:**
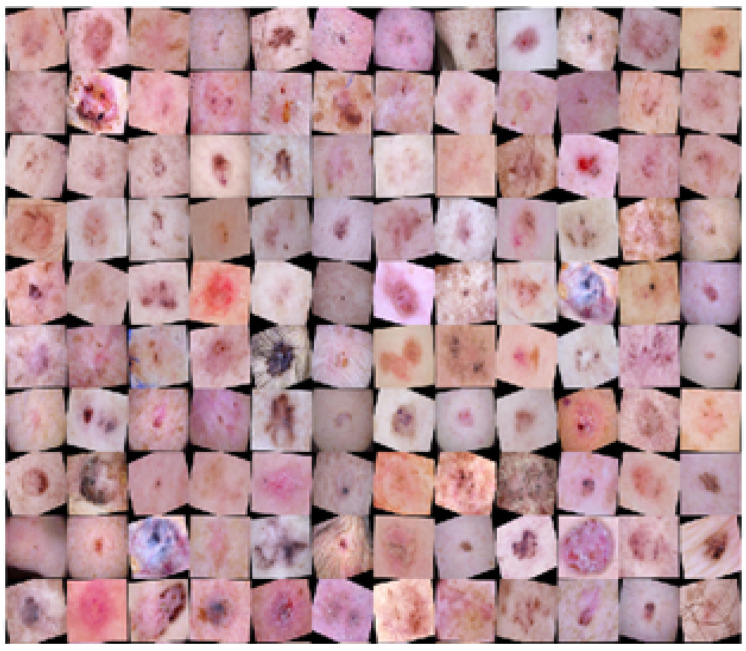
Augmented data samples using translation, rotation, and scaling.

The ISIC datasets still suffer from imbalance problems after using additional lesion samples because the data are highly skewed among several types of skin cancer. The data imbalance problem causes the network to become biased towards classes with many samples compared with those with low samples. The datasets were highly imbalanced; for example, the number of MEL samples was more than BEN in ISIC 2016, while that of BEN samples was more than MEL in the ISIC 2017 and 2020 sets. Table 1, Table 2 and Table 3 illustrate the distribution of data samples among different classes for three datasets. A few classes, such as the 512 MEL samples in the ISIC 2016, the 1214 MEL in the ISIC 2017, and the 3479 MEL samples in the ISIC 2020, were extended by generating more artificial samples using the random oversampling method. To address the issues of data undersampling, skewness, and image sample scarcity, data augmentation techniques were applied more to the underrepresented classes than oversampled classes. Moreover, online data augmentation was applied during network training using three common operations: rotation from −300 to +300, scaling with factors 0.8 in the X-direction and 1.0 in the Y-direction, and translation by −5 and +5. These operations were only applied on the training sets, whereas validation and test sets were not augmented and their original data distribution was used during the validation and testing processes. Figure 2 shows the augmented samples for the classes MEL and BEN.

### 3.5. Proposed DCNN Model

**Figure 3:**
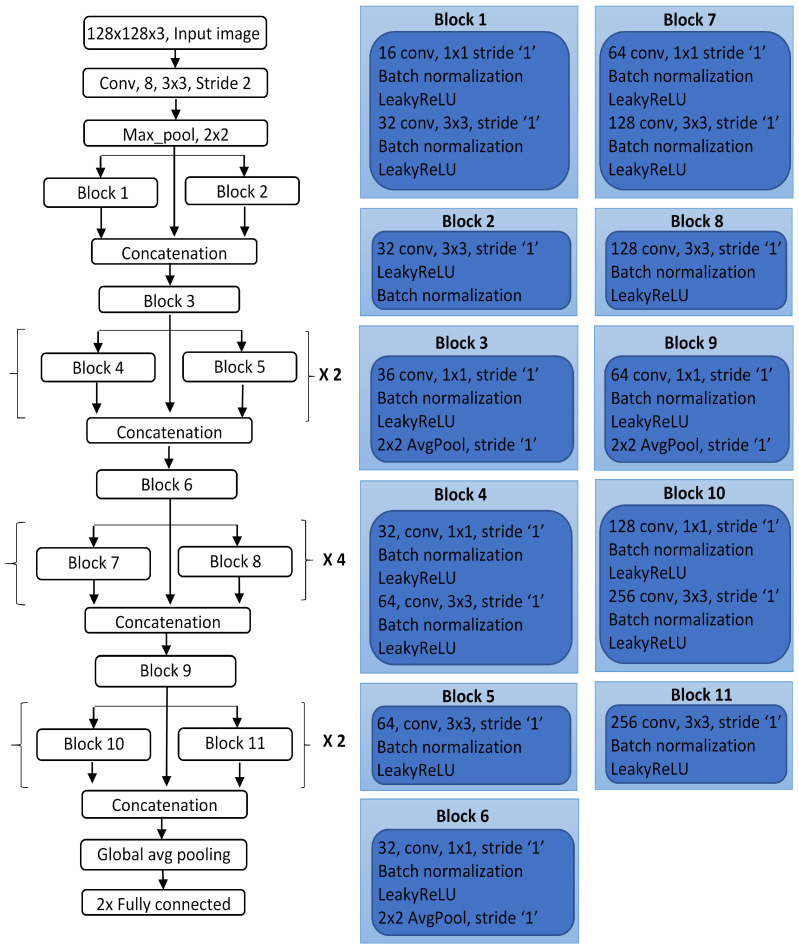
The design of the proposed network, LCNet.

The architecture of DCNN, named the lesion classification network (LCNet) is designed using 11 blocks organized as shown in Figure 3. The blocks 4 and 5, 7 and 8, and 10 and 11 are repeated at rates of 2, 4, and 2, respectively, from top to bottom to develop a deep layered network. The network’s first layer accepts an input image dataset of 128×128 with R, G, and B channels, followed by a convolutional operation that slides ‘8’ kernels of size 3×3 over an image with a stride value ‘2’. The primary purpose of this layer is to calculate features, and to do so, a small matrix called a kernel slides over an image and transforms the pixels’ values as follows:

(4) $$Conv\left[x,y\right]=\sum_{i=1}^{s}\sum_{j=1}^{s}\left(I_{x-i,y-j}*K_{i,j},\;n_{f}\right)$$

where Conv[x,y] is the output of the convolution operation for pixel positions [x,y] in the spatial domain, *s* is the kernel size, *I* is the input image, and *K* is the kernel or template with multiple channels $n_{f}$.

The output of this layer is in the form of a feature map that is passed to the next layer, i.e., max-pooling to transform the feature map regions by taking their maximum value. Pooling helps in reducing the size of feature maps. Each block consecutively uses three main layers: convolutional, batch normalization (BN), and leakyReLU. The input feature maps from previous layers are normalized using the batch normalization process in batches. It regulates the learning process of the network and avoids overfitting problems. The activation function used is leakyReLU, which offers the advantage of having a slight slope for negative values instead of the zero slope of the standard ReLU. The LeakyReLU function transforms negative values to positive by multiplying with a scalar value ‘s=0.3’ as:

(5) $$leakyReLU=\left\{\begin{array}{c} x\times s,\;x<0\\ x,\;\;\;\;x\geq 0 \end{array}\right.$$

Block 1 is composed of convolutional, BN, and leakyReLU layers used twice. The first convolutional layer contains ‘16’ kernels of size 1×1, and the second has ‘32’ kernels of size 3×3 with a stride of ‘1’. A stride is defined as the number of steps to slide the filter map on an image. As for block 2, it contains three layers as convolutional with ‘32’ filters having the size of 3×3 followed by leakyReLU and BN layer. The feature sets computed by blocks 1 and 2 and the pooling indices from max-pooling are concatenated to form a combined feature set, which then passes to block 3. Blocks 4, 7, and 10 follow a similar pattern to block 1, except the number of kernels increases from 32, 64, 64, 128, 128, and 256. The number of filters in blocks 5, 8, and 11 increased to 64, 128, and 256 of size 3×3 in the successive convolutional layers. Finally, in blocks 3, 6, and 9, the number of filters varies as 36, 32, and 64 of size 1×1 followed by an average poling layer. This block used an average pooling operation instead of max-pooling to calculate the average for each patch of the feature map that overlaps the filter window. This layer downsamples to an average value in the window with a filter size of 2×2.

The blocks are repeated to form a deep network to extract lesion information such as edges, colors, and complex lesion patterns in the form of a feature map. A global average pooling and fully connected layer are used at the end of the network for generating a single feature vector corresponding to each class category. The softmax function calculates the confidence score for interpreting the probability of falling into one of the given classes. The number of learnable parameters and kernels generated by the proposed network is less than other state-of-the-art networks, making it less complex and lightweight. For example, the total learnable parameters and number of kernels used in the studies [23,37,38,39] were 256.7 M and 267.5 M, 4.8 M and 45.6 M, 58.3 K and 84.7 K, and 4.6 K, and 29.1 K respectively. In contrast, the LCNet achieved high performances by optimizing the parameters and kernels as 3.3 M and 3.1 K, respectively.

The proposed deep neural model was inspired by many advanced frameworks [23,40] specifically designed to classify skin lesions, which is a challenging task for clinical experts to address in actual practice. Similar to these networks, the idea of designing a network with multiple blocks to form a DCNN is incorporated. However, there are many architectural differences between the proposed architecture and DCNN in [23,40]. The proposed network has a different number of layers, kernel size, and number of kernels used at each convolutional and max-pooling layer. As opposed to the model presented in [23], all blocks were sequentially repeated in the ratio of 2:4:2, forming a network with a total of 31 convolutional layers, which is fewer than the network presented by Iqbal et al. Furthermore, in our case, a different number of kernels was used in each convolutional layer in all blocks, whereas in the network given in /citeiqbal2021automated, each block had a fixed number of filters. Unlike the model presented by M.S. Ali et al. in [40], the LCNet makes use of multiple blocks and utilizes information from multiple channels by concatenating the features of each block to pass information to the next. Alternatively, the model presented by M. S. Ali used five blocks serially connected, followed by dropout and a fully connected layer.

A deep neural network generally suffers from computational cost and limited memory issues. Thus, the original images are rescaled to lower dimensions to tackle this issue. This rescaling operation ensures that contextual information about lesions is not lost for a skin classification task. Additionally, the skewed distribution of lesion samples is handled using augmentation operations and the random oversampling method. This method creates more samples in underrepresented classes to balance the distribution. The presence of noise artefacts and a high ratio of inter-class similarities and intra-class differences make the classification process highly challenging. Therefore, the proposed network with 31 convolutional layers can efficiently extract low- to high-level information. The network weights are optimized using the backpropagation algorithm that reduces loss based on the gradient value. It uses a stochastic gradient optimizer (SGDM) [41] to update the network weights and biases to reduce the loss value by applying small changes in the direction of optimization.

(6) $$\theta_{i+1}=\theta_{i}-\alpha ▽L\left(\theta_{i}\right)$$

where the number of iterations represented as *i*, α>0 is the learning parameter (set as ‘0.001’), θ is a parameter vector, and $▽L(\theta_{i})$ is the gradient of the loss function. At each iteration, the algorithm evaluates the gradient and updates parameters over a mini-batch set. The larger weight values can cause a network to be stuck in the local minima. Thus, the momentum γ is added in the gradient descent algorithm to reduce the oscillations as follows:

(7) $$\theta_{i+1}=\theta_{i}-\alpha ▽L\left(\theta_{i}\right)+\gamma \left(\theta_{i}+\theta_{i+1}\right)$$

Furthermore, the LCNet utilizes a cross-entropy loss [42] function that measures the error between the prediction score P and target T. The weighted cross-entropy loss function calculates the error as follows:

(8) $$Loss=\frac{1}{N}\sum_{i=1}^{K}\sum_{j=1}^{N}w_{i}T_{i_{j}}log\left(P_{i_{j}}\right)$$

where the number of observations is presented as *N*, *K* is the number of classes, and *w* is a vector of weights determined by the network for each class. The hyperparameters used for the LCNet are summarized in Table 4.

## 4. Results and Discussion

Several experiments for skin lesion classification were conducted on different dermoscopic lesion images to evaluate the performance of the LCNet. It was tested on three different sets, the ISIC 2016, ISIC 2017, ISIC 2020, and $PH^{2}$ for two classes, MEL and BEN. Other state-of-the-art methods depend highly on the noise removal preprocessing steps and region of interest (ROI) specific feature calculation for achieving a high classification rate. In contrast, the LCNet does not require extensive preprocessing operations and extraction of lesion features. It is trained end-to-end on dermoscopic images to distinguish melanoma and other lesion types. The hyperparameters (see Table 4) are finalized after several experiments and monitoring the network’s highest performance on the validation data. The network training was performed on the hardware configuration of GeForce GTX 1080 Ti with a computation capacity of ‘7.5’. Moreover, the inference time on ISIC 2016 with 344 test images was 3.77 s, that on ISIC 2017 with 835 test images was 15.7 s, and that on ISIC 2020 with 2014 test images was 61.6 s. Various classification performance metrics such as precision (PRE), recall (REC), accuracy (ACC), specificity (SPE), F1-Score, [43,44], and learnable parameters were considered to evaluate the model. The mathematical formulas used to calculate the values of these metrics are given as follows:

(9) $$ACC=\frac{TP+TN}{TP+FP+TN+FN}$$

(10) $$PRE=\frac{TP}{TP+FP}$$

(11) $$REC=\frac{TP}{TP+FN}$$

(12) $$SPE=\frac{TN}{TN+FP}$$

(13) $$F1\text{-}Score=\frac{2TP}{2TP+FP+FN}$$

In a confusion matrix, TP, FP, TN, and FN represent true positives, false positives, true negatives, and false negatives. TP represents the number of lesion samples correctly classified as melanoma, TN represents the number of lesion samples correctly classified as benign, FP represents the ratio of samples incorrectly classified as melanoma, and FN represents the images determined to be benign when they are melanoma. An ACC is defined as the fraction of correctly identified samples and the total number of predictions based on these parameters. Other parameters, PRE and REC, are very significant metrics used to evaluate the model’s performance as PRE measures all positive predicted rates. In contrast, REC calculates the true positive ratio out of all positively identified samples. The model’s ability to identify TN of each class is measured by a metric called SPE. Lastly, F1-Score measures the harmonic mean of PRE and REC by considering FP and FN. Its value close to 1 indicates the perfect PRE and REC.

The scarcity of lesion samples in different classes prevented bias using data augmentation and oversampling methods. The impact of using data oversampling on the network’s performance is shown in Table 5, which explains that there is an increase in the values of metrics on all datasets, where a drastic change is noticed on ISIC 2017. The reason for this is that the original ISIC 2017 dataset was highly imbalanced, giving poor results. In data extension, first, the training of the LCNet was performed on the augmented training set using the fine-tuned hyperparameters. The training progress was monitored on the validation set of the ISIC datasets. The validation set contains a different proportion of lesion samples from all classes, and the hyperparameters were tuned on the validation set to improve the performance. Thus, the final values were selected based on the best output offered by the network on the validation set having the lowest loss and high accuracy. Finally, the trained model with fine-tuned parameters was used to evaluate the test set unseen by the network.

**Figure 4:**
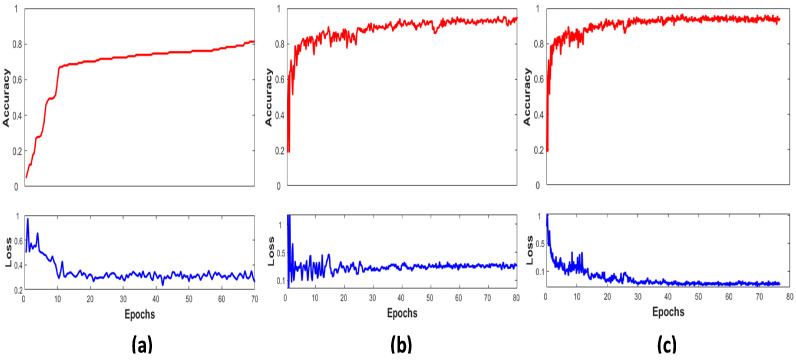
Classification accuracy and loss curves of the LCNet with the number of epochs on the validation set (**a**) MEL vs. BEN lesion classes ISIC 2016, (**b**) MEL vs. SK and NV lesion classes ISIC 2017, and (**c**) MEL vs. BEN lesion classes ISIC 2020.

Figure 4 shows the graphical view of the LCNet on the ISIC 2016, 2017, and 2020 validation sets by plotting their performance between accuracy and number of epochs. It displays the network’s accuracy progressively increasing towards higher values over the subsequent increase in the number of iterations per epochs. Early stopping criteria were implemented to stop the model’s training if accuracy did not improve and the corresponding loss did not decrease; hence, the LCNet converges after 80 epochs. The higher performance is noticed on the ISIC 2020 dataset due to a large number of samples present in it.

**Figure 5:**
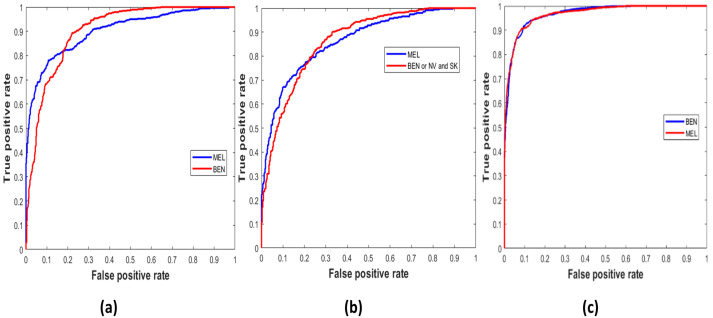
Classification accuracy and loss curves of the LCNet with the number of epochs on the validation set (**a**) MEL vs. BEN lesion classes ISIC 2016 (**b**) MEL vs. SK and NV lesion classes ISIC 2017 (**c**) MEL vs. BEN lesion classes ISIC 2020.

Similarly, Figure 5 demonstrates the true positive vs. false positive curves [45], illustrating the trade-off between sensitivity and specificity achieved by the model with the area under the curve (AUC) as ‘0.9033’, ‘0.8658’, and ’0.9671’ on the ISIC 2016, 2017, and 2020 test sets. In Table 6, the performance of the LCNet is illustrated on all datasets based on the classification metrics explained above. The LCNet model obtained ACC, PRE, and REC of 81.41%, 81.88%, and 81.30%, respectively, for the binary classification of MEL vs. BEN on the ISIC 2016 dataset. At the same time, the values for these metrics on the ISIC 2017 test set for the classification of classes, i.e., MEL vs. NV and SK, were 88.23%, 78.55%, and 87.86%, respectively. Furthermore, on the ISIC 2020 and $PH^{2}$ sets, the values for ACC, PRE, and REC achieved by the model were 90.42%, 90.48%, and 90.39% and 76.0%, 67.8%, and 75.3%, respectively. Moreover, the LCNet surpassed the other state-of-the-art approaches for skin lesion classification, as given in Table 7. It compares the methods with the best results highlighted in bold based on metrics such as ACC, PRE, REC, SPE, F1-Score, and learnable parameters. Only the ACC and SPE on the ISIC 2017 of the [46] were higher than in the proposed model, whereas the PRE of the LCNet is the highest among all given studies. In addition, the number of learnable parameters of LCNet is less, making it a lightweight and less complex network.

In Table 8, the performances of baseline CNN models such as ResNet18, Inceptionv3, and AlexNet are displayed. These popular networks were fine-tuned on the adopted datasets, and a comparison was shown between them and the LCNet model. For tuning them, the same hyperparameters setting were used as for the proposed model (see Table 4). It can be seen in Table 8 that the proposed model outperformed given networks. The metrics ACC, PRE, and REC represent the prediction score of the models on the ISIC 2016, 2017, and 2020 test sets, classifying lesion classes by giving better insight into correctly classified and misclassified samples based on the evaluation metrics. The proposed network achieved 0.5% more ACC than ResNet18, 1.5% than Inceptionv3, and 16% more than the AlexNet model on the ISIC 2016 dataset. Similarly, on the ISIC 2017 dataset, LCNet gained 13.2% higher ACC than ResNet18, 10.8% higher ACC than Inceptionv3, and 14.2% higher ACC than the AlexNet network. Lastly, the ACC of ResNet18 was slightly more than LCNet with a ratio of 0.4%, whereas LCNet outperformed Incpetionv3 and AlexNet by a higher margin. It is observed that the proposed LCNet model gained a higher accuracy on all datasets, which is higher among other popular models.

The experimental outcomes prove that the proposed model performs better for binary skin cancer classification tasks. PRE, REC, and ACC are relatively higher on the ISIC 2020 datasets. In contrast, these metrics observed lower values on ISIC 2017 and 2016 than the ISIC 2020 due to the fewer samples in each class. It is analysed that the deep learning-based LCNet model requires a large dataset for efficient network training. The primary advantage of the proposed model is that the inference time is very low on test sets and have a smaller number of learnable parameters.

## 5. Conclusions

Skin cancer is a global health problem, and the development of an automatic melanoma detection system plays a major role in its early diagnosis. The proposed LCNet model, inspired by the deep convolutional neural network for skin cancer classification, was trained in an end-to-end manner on dermoscopic skin cancer images. Three different datasets from the ISIC challenge were incorporated to perform the experiments, and an additional $PH^{2}$ set was used for testing. It is challenging to establish an automatic framework to classify different lesions due to high inter-similarities and intra-class variations. With the design of a few preprocessing steps such as image resizing, oversampling, and augmentation, an accurate model was designed for MEL lesion classification. The experimental results showed that the proposed model achieved higher performance than the selected studies and pre-trained classification models. Overall, LCNet achieved average ACC, PRE, and REC of 81.41%, 81.88%, and 81.30% on ISIC 2016, of 88.23%, 78.55%, and 87.86% on ISIC 2017, and of 90.48%, 90.39%, and 90.42% on ISIC 2020. The proposed model is reliable in predicting the correct lesion category with a high true positive rate, thus strongly satisfying AI in solving medical problems as a diagnostic tool. It was found that using an image size of 128×128 with three channels and the inference time per image of 0.1 s could achieve a higher processing speed. Therefore, the proposed method could perform better on large and balanced skin cancer datasets, such as the ISIC 2020 dataset, compared with the ISIC 2016 and 2017. The designed DCNN model can be further extended to multi-class classification to predict other different types of skin cancers.

## Figures and Tables

**Table 1:** The ISIC 2016 data distribution among training, validation, and test sets.

Classes	Training Samples	Augmented Training	Validation Samples	Test Samples	Total Samples
	70%	Samples	10%	20%	100%
MEL	512	692	98	146	756
BEN	692	692	73	198	963
Total	1200	1384	171	344	1719

**Table 2:** The ISIC 2017 data distribution among training, validation, and test sets.

Classes	Training Samples	Augmented Training	Validation Samples	Test Samples	Total Samples
	70%	Samples	10%	20%	100%
MEL	1214	1708	173	347	1732
BEN	1708	1708	244	488	2440
Total	2922	3416	417	835	4172

**Table 3:** The ISIC 2020 data distribution among training, validation, and test sets.

Classes	Training Samples	Augmented Training	Validation Samples	Test Samples	Total Samples
	70%	Samples	10%	20%	100%
MEL	3479	3570	497	994	4970
BEN	3570	3570	510	1020	5100
Total	7049	7140	1007	2014	10070

**Table 4:** Hyperparameter selected for the proposed LCNet.

Learning Algorithm	Learning Rate	Mini-Batch Size	Epochs	Activation Function	Data Augmentation	Momentum	Regularization
SGDM	0.001	32	100	LeakyReLU	Random oversampling, rotation, translation, and scaling	0.99	0.0005

**Table 5:** Impact of data oversampling on the performance of LCNet.

Approach	ISIC 2016			ISIC 2017			ISIC 2020		
	ACC	PRE	REC	ACC	PRE	REC	ACC	PRE	REC
Without oversampling	0.773	0.779	0.765	0.607	0.529	0.518	0.886	0.874	0.896
With oversampling	0.814	0.818	0.813	0.882	0.785	0.878	0.904	0.904	0.903

**Table 6:** Performance of the LCNet on the adopted datasets.

ISIC 2016			ISIC 2017			ISIC 2020			${\mathrm{PH}}^{2}$		
ACC	PRE	REC	ACC	PRE	REC	ACC	PRE	REC	ACC	PRE	REC
0.814	0.818	0.813	0.882	0.785	0.878	0.904	0.904	0.903	0.760	0.678	0.753

**Table 7:** Performance comparison of LCNet with other state-of-the-art methods.

Methods/Authors	Dataset	ACC%	PRE%	REC%	SPE%	F-Score%	Learnable Parameters (Millions)
Al-Masni, M. A. [47]	ISIC 2016	81.79	—–	81.80	71.40	82.59	—–
Zhang J. [48]		86.28	68.10	—–	—–	—–	—–
Tang P. [46]		**86.30**	72.80	32.00	**99.70**	—–	—–
**Proposed model**		81.41	**81.88**	**81.30**	80.83	**81.05**	**3.32 M**
Mahbod, A. [37]	ISIC 2017	87.70	—–	87.26	82.18	—–	256.7 M
Harangi, B. [38]		86.60	—–	55.60	78.50	—–	267.5 M
Li, Y. et al. [21]		85.70	72.9	49.00	**96.10**	—–	—–
Al-Masni, M. A. [47]		81.34	75.67	77.66	75.72	—–	54.35 M
Iqbal, I. [23]		93.25	93.97	93.25	90.64	93.47	4.8M
**Proposed Model**		**88.23**	**78.55**	**87.86**	88.86	**78.20**	**3.32 M**
Kwasigroch, A. [49]	ISIC 2020	77.00	—–	—–	—–	—–	7.18 M
**Proposed Model**		**90.42**	**90.48**	**90.39**	**90.39**	**90.41**	**3.32 M**

**Table 8:** A comparison between proposed LCNet with baseline CNN models on the ISIC 2016, 2017, and 2020 datasets.

Approach	ISIC 2016			ISIC 2017			ISIC 2020		
	ACC	PRE	REC	ACC	PRE	REC	ACC	PRE	REC
ResNet18	0.809	0.789	0.809	0.750	0.640	0.571	0.908	0.898	0.888
Inceptionv3	0.799	0.809	0.811	0.774	0.691	0.612	0.486	0.297	0.492
AlexNet	0.654	0.595	0.643	0.740	0.670	0.660	0.754	0.691	0.685
**Proposed model (LCNet)**	**0.814**	**0.818**	**0.813**	**0.882**	**0.785**	**0.878**	**0.904**	**0.904**	**0.903**

## Data Availability

The data are publicly available at https://www.isic.org/.

## Abbreviations

DCNN	Deep convolutional neural network
ISIC	International skin imaging collaboration
ReLU	Rectified linear unit
BN	Batch normalization
